# Diaqua­(2,2′-bipyridine-5,5′-dicarboxyl­ato-κ^2^ *N*,*N*′)(ethyl­enediamine-κ^2^ *N*,*N*′)copper(II) 2.5-hydrate

**DOI:** 10.1107/S1600536808029061

**Published:** 2008-09-20

**Authors:** Mohammad Yousefi, Aida Khalighi, Nasim Tadayon Pour, Vahid Amani, Hamid Reza Khavasi

**Affiliations:** a Islamic Azad University, Shahr-e-Rey Branch, Tehran, Iran; b Department of Chemistry, Shahid Beheshti University, Tehran 1983963113, Iran

## Abstract

In the mol­ecule of the title compound, [Cu(C_12_H_6_N_2_O_4_)(C_2_H_8_N_2_)(H_2_O)_2_]·2.5H_2_O, the Cu^II^ atom is six-coordinated in a distorted octa­hedral configuration by two N atoms from a 2,2′-bipyridine-5,5′-dicarboxyl­ate anion, two N atoms from ethyl­enediamine and two O atoms from two water mol­ecules. There are also two and a half water mol­ecules in the asymmetric unit. The planar five-membered ring is nearly coplanar with the adjacent pyridine rings, while the other five-membered ring adopts a twisted conformation, probably due to hydrogen bonding. In the crystal structure, intra- and inter­molecular N—H⋯O and O—H⋯O hydrogen bonds link the mol­ecules.

## Related literature

For complexes involving 2,2′-bipyridine-5,5′-dicarboxyl­ate anions, see: Min *et al.* (2002 ▶); Geary *et al.* (2003 ▶); Hafizovic *et al.* (2006 ▶); Schoknechta & Kempe (2004 ▶); Matthews *et al.* (2004 ▶).
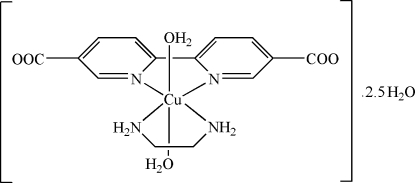


## Experimental

### 

#### Crystal data

[Cu(C_12_H_6_N_2_O_4_)(C_2_H_8_N_2_)(H_2_O)_2_]·2.5H_2_O
*M* *_r_* = 446.92
Monoclinic, 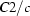

*a* = 31.730 (6) Å
*b* = 7.2481 (14) Å
*c* = 18.421 (4) Å
β = 120.05 (3)°
*V* = 3667.1 (17) Å^3^
*Z* = 8
Mo *K*α radiation
μ = 1.25 mm^−1^
*T* = 298 (2) K
0.50 × 0.18 × 0.07 mm

#### Data collection

Bruker SMART CCD area-detector diffractometer
Absorption correction: multi-scan (*SADABS*; Sheldrick, 1998 ▶) *T*_min_ = 0.770, *T*_max_ = 0.923
13747 measured reflections
4887 independent reflections
4221 reflections with *I* > 2σ(*I*)
*R*_int_ = 0.043

#### Refinement

*R*[*F* ^2^ > 2σ(*F* ^2^)] = 0.045
*wR*(*F* ^2^) = 0.120
*S* = 1.10
4887 reflections
301 parameters
H atoms treated by a mixture of independent and constrained refinement
Δρ_max_ = 1.71 e Å^−3^
Δρ_min_ = −0.64 e Å^−3^

### 

Data collection: *SMART* (Bruker, 1998 ▶); cell refinement: *SAINT* (Bruker, 1998 ▶); data reduction: *SAINT*; program(s) used to solve structure: *SHELXTL* (Sheldrick, 2008 ▶); program(s) used to refine structure: *SHELXTL*; molecular graphics: *ORTEP-3 for Windows* (Farrugia, 1997 ▶); software used to prepare material for publication: *WinGX* (Farrugia, 1999 ▶).

## Supplementary Material

Crystal structure: contains datablocks I, global. DOI: 10.1107/S1600536808029061/hk2516sup1.cif

Structure factors: contains datablocks I. DOI: 10.1107/S1600536808029061/hk2516Isup2.hkl

Additional supplementary materials:  crystallographic information; 3D view; checkCIF report

## Figures and Tables

**Table:** 

O5—Cu1	2.563 (3)
O6—Cu1	2.499 (3)
N1—Cu1	2.018 (2)
N2—Cu1	2.0225 (19)
N3—Cu1	2.003 (2)
N4—Cu1	2.015 (2)

**Table:** 

O5—Cu1—O6	174.49 (10)
O5—Cu1—N1	86.22 (11)
O5—Cu1—N2	88.78 (10)
O5—Cu1—N3	90.26 (10)
O5—Cu1—N4	89.86 (11)
O6—Cu1—N1	89.50 (10)
O6—Cu1—N2	93.97 (10)
O6—Cu1—N3	86.84 (10)
O6—Cu1—N4	94.56 (11)
N3—Cu1—N4	85.24 (9)
N3—Cu1—N1	97.29 (9)
N4—Cu1—N1	175.34 (9)
N3—Cu1—N2	177.97 (9)
N4—Cu1—N2	96.54 (8)
N1—Cu1—N2	80.87 (8)

**Table 2:** Hydrogen-bond geometry (Å, °)

*D*—H⋯*A*	*D*—H	H⋯*A*	*D*⋯*A*	*D*—H⋯*A*
N3—H3*A*⋯O2^i^	0.84 (3)	2.11 (3)	2.881 (3)	153 (4)
N3—H3*B*⋯O3^ii^	0.86 (4)	2.21 (4)	3.031 (3)	159 (4)
N4—H4*B*⋯O8	0.87 (3)	2.20 (3)	3.054 (3)	171 (4)
N4—H4*C*⋯O9^iii^	0.89 (4)	2.23 (4)	3.069 (4)	158 (4)
O5—H5*B*⋯O7^iv^	0.86 (6)	1.97 (6)	2.807 (4)	164 (5)
O5—H5*C*⋯O9^ii^	0.72 (7)	2.08 (6)	2.779 (5)	165 (5)
O6—H6*A*⋯O1^i^	0.74 (5)	1.99 (5)	2.726 (4)	171 (4)
O6—H6*B*⋯O3^iii^	0.95 (7)	2.51 (7)	3.267 (4)	136 (5)
O7—H7*A*⋯O2	0.82 (6)	2.00 (6)	2.776 (5)	158 (5)
O7—H7*B*⋯O4^v^	0.78 (7)	2.11 (7)	2.827 (4)	153 (7)
O8—H8*B*⋯O7^iv^	0.76 (6)	2.22 (6)	2.969 (5)	178 (8)
O9—H9*B*⋯O4^vi^	0.86 (6)	1.96 (5)	2.744 (4)	152 (5)
O9—H9*C*⋯O3	0.97 (6)	1.74 (6)	2.706 (3)	169 (4)

Symmetry codes: (i) 
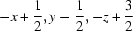
; (ii) 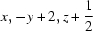; (iii) 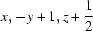; (iv) 
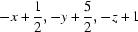
; (v) 
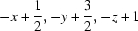
; (vi) 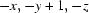.

**Fig. 1.**
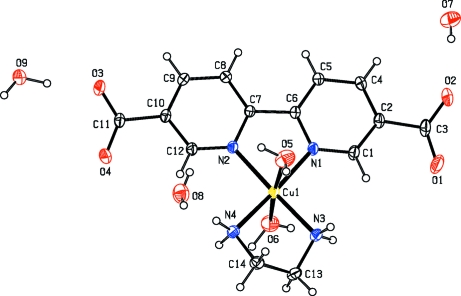
The molecular structure of the title molecule, with the atom-numbering scheme. Displacement ellipsoids are drawn at the 40% probability level.

**Fig. 2.**
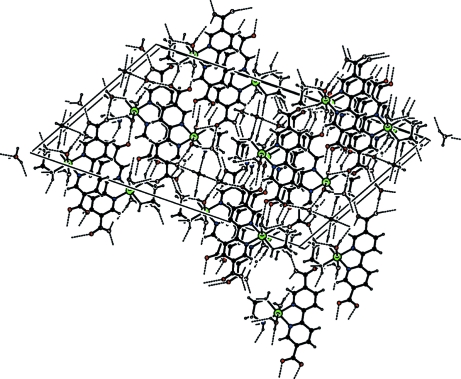
A packing diagram of the title compound. Hydrogen bonds are shown as dashed lines.

## supplementary crystallographic information

### Comment

2,2'-Bipyridine-5,5'-dicarboxylic acid (BPDCH_2_) is a good bridging ligand, and numerous complexes with BPDCH_2_ anions have been prepared, such as that of cobalt (Min *et al.*,  2002), platinum (Geary *et al.*, 2003; Hafizovic *et al.*,  2006), neodyminum (Schoknechta & Kempe, 2004), ruthenium and rhodium (Matthews *et al.*, 2004) complexes. For further investigation of 2,2'-bipyridine-5,5'-dicarboxylic acid, we synthesized the title compound and report herein its crystal structure.

In the title compound, (Fig. 1), the Cu^II^ atom is six-coordinated in a distorted octahedral configuration by two N atoms from 2,2'-bipyridine-5,5'-dicarboxylate anion, two N atoms from ethylenediamine and two O atoms from two water molecules (Table 1). There are also two and a half water molecules in the asymmetric unit. Rings A (N1/C1/C2/C4–C6), B (N2/C7–C10/C12) and C (Cu1/N1/N2/C6/C7) are, of course, planar, and the dihedral angles between them are A/B = 1.43 (3)°, A/C = 2.09 (3)° and B/C = 2.45 (3)°. So, they are nearly coplanar, while ring D (Cu1/N3/N4/C13/C14) adopts twisted conformation, probably due to the hydrogen bondings.

In the crystal structure, intra- and intermolecular N—H···O and O—H···O hydrogen bonds (Table 2) link the molecules (Fig. 2), in which they may be effective in the stabilization of the structure.

### Experimental

For the preparation of the title compound, ethylenediamine (0.15 g, 2.50 mmol) was added to a suspension of 2,2'-bipyridine-5,5'-dicarboxylic acid (0.21 g, 0.83 mmol) in water (10 ml) and the resulting colorless solution was added to CuCl_2_.2H_2_O (0.14 g, 0.83 mmol) in water (10 ml). The resulting blue solution was stirred at 323 K for 15 min, and then was left to evaporate slowly at room temperature. After one week, blue plate crystals of the title compound were isolated (yield; 0.26 g, 70.01%, m.p. 488 K).

### Refinement

H3A, H3B, H4B, H4C (for NH_2_) and H5B, H5C, H6A, H6B, H7A, H7B, H8B, H9B, H9C (for H_2_O) atoms were located in difference syntheses and refined isotropically [N—H = 0.85 (4)–0.89 (4) Å and U_iso_(H) = 0.035 (8)–0.043 (9) Å^2^; O—H = 0.72 (5)–0.97 (6) Å and U_iso_(H) = 0.041 (10)–0.11 (2) Å^2^]. The remaining H atoms were positioned geometrically, with C—H = 0.93 and 0.97 Å for aromatic and methylene H, respectively, and constrained to ride on their parent atoms with U_iso_(H) = 1.2U_eq_(C).

### Crystal data

**Table:**

[Cu(C_12_H_6_N_2_O_4_)(C_2_H_8_N_2_)(H_2_O)_2_]·2.5H_2_O	*F*(000) = 1856
*M**_r_* = 446.92	*D*_x_ = 1.619 Mg m^−^^3^
Monoclinic, *C*2/*c*	Mo *K*α radiation, λ = 0.71073 Å
Hall symbol: -C 2yc	Cell parameters from 1976 reflections
*a* = 31.730 (6) Å	θ = 2.2–29.3°
*b* = 7.2481 (14) Å	µ = 1.25 mm^−^^1^
*c* = 18.421 (4) Å	*T* = 298 K
β = 120.05 (3)°	Plate, blue
*V* = 3667.1 (17) Å^3^	0.50 × 0.18 × 0.07 mm
*Z* = 8	

### Data collection

**Table:**

Bruker SMART CCD area-detector diffractometer	4887 independent reflections
Radiation source: fine-focus sealed tube	4221 reflections with *I* > 2σ(*I*)
graphite	*R*_int_ = 0.043
φ and ω scans	θ_max_ = 29.3°, θ_min_ = 2.2°
Absorption correction: multi-scan (SADABS; Sheldrick, 1998)	*h* = −43→43
*T*_min_ = 0.770, *T*_max_ = 0.923	*k* = −9→9
13747 measured reflections	*l* = −25→25

### Refinement

**Table:**

Refinement on *F*^2^	Primary atom site location: structure-invariant direct methods
Least-squares matrix: full	Secondary atom site location: difference Fourier map
*R*[*F*^2^ > 2σ(*F*^2^)] = 0.045	Hydrogen site location: inferred from neighbouring sites
*wR*(*F*^2^) = 0.120	H atoms treated by a mixture of independent and constrained refinement
*S* = 1.10	*w* = 1/[σ^2^(*F*_o_^2^) + (0.0582*P*)^2^ + 5.7488*P*] where *P* = (*F*_o_^2^ + 2*F*_c_^2^)/3
4887 reflections	(Δ/σ)_max_ = 0.015
301 parameters	Δρ_max_ = 1.71 e Å^−^^3^
0 restraints	Δρ_min_ = −0.64 e Å^−^^3^

### Special details

**Table:**

Geometry. All e.s.d.'s (except the e.s.d. in the dihedral angle between two l.s. planes) are estimated using the full covariance matrix. The cell e.s.d.'s are taken into account individually in the estimation of e.s.d.'s in distances, angles and torsion angles; correlations between e.s.d.'s in cell parameters are only used when they are defined by crystal symmetry. An approximate (isotropic) treatment of cell e.s.d.'s is used for estimating e.s.d.'s involving l.s. planes.
Refinement. Refinement of *F*^2^ against ALL reflections. The weighted *R*-factor *wR* and goodness of fit *S* are based on *F*^2^, conventional *R*-factors *R* are based on *F*, with *F* set to zero for negative *F*^2^. The threshold expression of *F*^2^ > σ(*F*^2^) is used only for calculating *R*-factors(gt) *etc*. and is not relevant to the choice of reflections for refinement. *R*-factors based on *F*^2^ are statistically about twice as large as those based on *F*, and *R*- factors based on ALL data will be even larger.

### Fractional atomic coordinates and isotropic or equivalent isotropic displacement parameters (Å^2^)

**Table:**

	*x*	*y*	*z*	*U*_iso_*/*U*_eq_	
Cu1	0.129145 (10)	0.87872 (5)	0.482858 (17)	0.02876 (10)	
O1	0.28103 (9)	1.1389 (6)	0.78353 (15)	0.0769 (10)	
O2	0.33829 (8)	1.2562 (4)	0.76199 (13)	0.0501 (5)	
O3	0.09405 (8)	0.6749 (3)	0.10781 (12)	0.0447 (5)	
O4	0.05397 (7)	0.5529 (3)	0.16672 (12)	0.0410 (4)	
O5	0.10527 (11)	1.2087 (4)	0.42720 (19)	0.0570 (6)	
H5B	0.0955 (18)	1.190 (7)	0.375 (4)	0.078 (15)*	
H5C	0.0838 (19)	1.250 (8)	0.424 (3)	0.081 (18)*	
O6	0.15861 (10)	0.5703 (4)	0.55005 (19)	0.0513 (6)	
H6A	0.1748 (14)	0.577 (5)	0.596 (3)	0.041 (10)*	
H6B	0.133 (2)	0.484 (9)	0.535 (4)	0.101 (19)*	
O7	0.41911 (13)	1.2811 (5)	0.74176 (19)	0.0621 (7)	
H7A	0.3946 (18)	1.303 (7)	0.744 (3)	0.062 (13)*	
H7B	0.434 (2)	1.192 (10)	0.764 (4)	0.11 (2)*	
O8	0.0000	0.9968 (6)	0.2500	0.0568 (9)	
H8B	0.0210 (17)	1.050 (7)	0.252 (4)	0.066 (15)*	
O9	0.03530 (9)	0.5945 (4)	−0.05647 (15)	0.0511 (6)	
H9B	0.0049 (17)	0.569 (6)	−0.080 (3)	0.063 (12)*	
H9C	0.056 (2)	0.608 (7)	0.004 (4)	0.097 (17)*	
N1	0.19871 (7)	0.9679 (3)	0.53881 (12)	0.0270 (4)	
N2	0.14333 (7)	0.8272 (3)	0.38928 (12)	0.0252 (4)	
N3	0.11694 (8)	0.9366 (3)	0.57706 (13)	0.0274 (4)	
H3A	0.1385 (13)	0.896 (5)	0.624 (2)	0.043 (9)*	
H3B	0.1155 (12)	1.055 (5)	0.581 (2)	0.035 (8)*	
N4	0.05847 (7)	0.8043 (3)	0.41939 (14)	0.0300 (4)	
H4B	0.0422 (13)	0.848 (5)	0.369 (2)	0.037 (8)*	
H4C	0.0557 (12)	0.682 (5)	0.418 (2)	0.037 (8)*	
C1	0.22492 (9)	1.0341 (4)	0.61691 (15)	0.0331 (5)	
H1	0.2111	1.0336	0.6511	0.040*	
C2	0.27180 (8)	1.1035 (3)	0.64927 (15)	0.0298 (5)	
C3	0.29927 (10)	1.1729 (4)	0.73932 (16)	0.0384 (6)	
C4	0.29229 (8)	1.0998 (3)	0.59865 (15)	0.0290 (5)	
H4A	0.3235	1.1457	0.6183	0.035*	
C5	0.26621 (8)	1.0276 (3)	0.51827 (14)	0.0271 (4)	
H5A	0.2800	1.0220	0.4841	0.033*	
C6	0.21916 (8)	0.9637 (3)	0.48967 (13)	0.0226 (4)	
C7	0.18787 (8)	0.8846 (3)	0.40512 (13)	0.0223 (4)	
C8	0.20220 (8)	0.8669 (3)	0.34572 (14)	0.0279 (4)	
H8A	0.2328	0.9074	0.3573	0.033*	
C9	0.17036 (9)	0.7882 (3)	0.26864 (14)	0.0284 (5)	
H9A	0.1795	0.7759	0.2281	0.034*	
C10	0.12496 (8)	0.7278 (3)	0.25224 (14)	0.0257 (4)	
C11	0.08798 (9)	0.6439 (3)	0.16880 (14)	0.0293 (5)	
C12	0.11346 (8)	0.7489 (4)	0.31536 (14)	0.0287 (5)	
H12	0.0834	0.7063	0.3056	0.034*	
C13	0.06969 (9)	0.8537 (4)	0.55719 (16)	0.0340 (5)	
H13A	0.0571	0.9136	0.5895	0.041*	
H13B	0.0738	0.7234	0.5711	0.041*	
C14	0.03498 (9)	0.8794 (4)	0.46480 (16)	0.0332 (5)	
H14A	0.0047	0.8148	0.4482	0.040*	
H14B	0.0278	1.0093	0.4520	0.040*	

### Atomic displacement parameters (Å^2^)

**Table:**

	*U*^11^	*U*^22^	*U*^33^	*U*^12^	*U*^13^	*U*^23^
Cu1	0.02016 (14)	0.04566 (19)	0.01986 (14)	−0.00232 (11)	0.00958 (11)	−0.00645 (12)
O1	0.0448 (13)	0.154 (3)	0.0339 (11)	−0.0239 (16)	0.0215 (10)	−0.0362 (16)
O2	0.0412 (11)	0.0628 (14)	0.0320 (10)	−0.0159 (10)	0.0077 (9)	−0.0168 (10)
O3	0.0551 (12)	0.0527 (12)	0.0221 (8)	−0.0170 (10)	0.0163 (8)	−0.0079 (8)
O4	0.0333 (9)	0.0507 (11)	0.0298 (9)	−0.0135 (8)	0.0090 (8)	−0.0066 (8)
O5	0.0548 (15)	0.0671 (17)	0.0513 (15)	0.0147 (13)	0.0281 (13)	0.0149 (13)
O6	0.0509 (14)	0.0504 (13)	0.0545 (15)	0.0021 (11)	0.0278 (13)	0.0043 (12)
O7	0.0718 (19)	0.0669 (18)	0.0573 (16)	0.0017 (15)	0.0396 (15)	0.0151 (14)
O8	0.054 (2)	0.057 (2)	0.0453 (19)	0.000	0.0144 (18)	0.000
O9	0.0465 (12)	0.0693 (16)	0.0318 (10)	−0.0222 (11)	0.0153 (9)	−0.0088 (10)
N1	0.0205 (8)	0.0369 (10)	0.0204 (8)	−0.0018 (7)	0.0079 (7)	−0.0056 (8)
N2	0.0213 (8)	0.0325 (10)	0.0206 (8)	−0.0014 (7)	0.0096 (7)	−0.0041 (7)
N3	0.0262 (9)	0.0355 (11)	0.0206 (9)	0.0039 (8)	0.0118 (8)	0.0020 (8)
N4	0.0237 (9)	0.0398 (12)	0.0253 (9)	−0.0018 (8)	0.0115 (8)	−0.0014 (9)
C1	0.0244 (10)	0.0483 (14)	0.0233 (10)	−0.0004 (10)	0.0095 (9)	−0.0095 (10)
C2	0.0249 (10)	0.0344 (12)	0.0219 (10)	0.0017 (9)	0.0056 (8)	−0.0048 (9)
C3	0.0298 (12)	0.0499 (15)	0.0248 (11)	0.0014 (11)	0.0058 (9)	−0.0130 (11)
C4	0.0240 (10)	0.0298 (11)	0.0262 (10)	−0.0037 (8)	0.0073 (8)	−0.0034 (9)
C5	0.0253 (10)	0.0309 (11)	0.0228 (10)	−0.0025 (8)	0.0103 (8)	−0.0013 (9)
C6	0.0226 (9)	0.0227 (10)	0.0197 (9)	0.0010 (8)	0.0085 (8)	−0.0014 (8)
C7	0.0218 (9)	0.0238 (9)	0.0194 (9)	0.0003 (8)	0.0088 (8)	0.0002 (8)
C8	0.0268 (10)	0.0336 (11)	0.0241 (10)	−0.0054 (9)	0.0133 (9)	−0.0024 (9)
C9	0.0325 (11)	0.0333 (12)	0.0211 (10)	−0.0023 (9)	0.0147 (9)	−0.0014 (9)
C10	0.0285 (10)	0.0266 (10)	0.0184 (9)	0.0006 (8)	0.0090 (8)	−0.0009 (8)
C11	0.0330 (11)	0.0280 (11)	0.0199 (9)	−0.0015 (9)	0.0079 (9)	−0.0029 (8)
C12	0.0236 (10)	0.0375 (12)	0.0230 (10)	−0.0036 (9)	0.0102 (8)	−0.0057 (9)
C13	0.0332 (12)	0.0447 (14)	0.0304 (12)	0.0028 (10)	0.0207 (10)	0.0034 (10)
C14	0.0238 (10)	0.0434 (13)	0.0333 (12)	0.0025 (10)	0.0150 (9)	0.0015 (11)

### Geometric parameters (Å, °)

**Table:**

O5—Cu1	2.563 (3)	C4—C5	1.387 (3)
O5—H5B	0.86 (6)	C4—H4A	0.9300
O5—H5C	0.72 (5)	C5—C6	1.390 (3)
O6—Cu1	2.499 (3)	C5—H5A	0.9300
O6—H6A	0.74 (4)	C6—N1	1.353 (3)
O6—H6B	0.96 (6)	C6—C7	1.481 (3)
O7—H7A	0.81 (5)	C7—N2	1.357 (3)
O7—H7B	0.78 (7)	C7—C8	1.385 (3)
O8—H8B	0.76 (6)	C8—C9	1.388 (3)
O9—H9B	0.86 (5)	C8—H8A	0.9300
O9—H9C	0.97 (6)	C9—C10	1.386 (3)
N1—Cu1	2.018 (2)	C9—H9A	0.9300
N2—Cu1	2.0225 (19)	C10—C12	1.390 (3)
N3—Cu1	2.003 (2)	C10—C11	1.518 (3)
N3—H3A	0.85 (4)	C11—O4	1.249 (3)
N3—H3B	0.86 (4)	C11—O3	1.252 (3)
N4—Cu1	2.015 (2)	C12—N2	1.335 (3)
N4—H4B	0.87 (4)	C12—H12	0.9300
N4—H4C	0.89 (4)	C13—N3	1.481 (3)
C1—N1	1.339 (3)	C13—C14	1.506 (4)
C1—C2	1.390 (3)	C13—H13A	0.9700
C1—H1	0.9300	C13—H13B	0.9700
C2—C4	1.378 (3)	C14—N4	1.475 (3)
C2—C3	1.522 (3)	C14—H14A	0.9700
C3—O1	1.237 (4)	C14—H14B	0.9700
C3—O2	1.247 (4)		
			
O5—Cu1—O6	174.49 (10)	C4—C2—C1	118.0 (2)
O5—Cu1—N1	86.22 (11)	C4—C2—C3	122.5 (2)
O5—Cu1—N2	88.78 (10)	C1—C2—C3	119.5 (2)
O5—Cu1—N3	90.26 (10)	O1—C3—O2	126.1 (3)
O5—Cu1—N4	89.86 (11)	O1—C3—C2	116.8 (3)
O6—Cu1—N1	89.50 (10)	O2—C3—C2	117.1 (3)
O6—Cu1—N2	93.97 (10)	C2—C4—C5	119.9 (2)
O6—Cu1—N3	86.84 (10)	C2—C4—H4A	120.1
O6—Cu1—N4	94.56 (11)	C5—C4—H4A	120.1
N3—Cu1—N4	85.24 (9)	C4—C5—C6	118.9 (2)
N3—Cu1—N1	97.29 (9)	C4—C5—H5A	120.5
N4—Cu1—N1	175.34 (9)	C6—C5—H5A	120.5
N3—Cu1—N2	177.97 (9)	N1—C6—C5	121.5 (2)
N4—Cu1—N2	96.54 (8)	N1—C6—C7	114.76 (18)
N1—Cu1—N2	80.87 (8)	C5—C6—C7	123.7 (2)
H5B—O5—H5C	101 (5)	N2—C7—C8	121.3 (2)
H6A—O6—H6B	112 (5)	N2—C7—C6	115.00 (18)
H7A—O7—H7B	118 (6)	C8—C7—C6	123.65 (19)
H9B—O9—H9C	123 (4)	C7—C8—C9	119.2 (2)
Cu1—O5—H5B	100 (3)	C7—C8—H8A	120.4
Cu1—O5—H5C	120 (5)	C9—C8—H8A	120.4
H5B—O5—H5C	100 (6)	C10—C9—C8	119.8 (2)
Cu1—O6—H6B	113 (4)	C10—C9—H9A	120.1
H6A—O6—H6B	112 (5)	C8—C9—H9A	120.1
Cu1—O6—H6A	112 (3)	C9—C10—C12	117.5 (2)
C1—N1—C6	118.6 (2)	C9—C10—C11	122.6 (2)
C1—N1—Cu1	126.54 (17)	C12—C10—C11	119.8 (2)
C6—N1—Cu1	114.80 (14)	O4—C11—O3	125.9 (2)
C12—N2—C7	118.72 (19)	O4—C11—C10	117.5 (2)
C12—N2—Cu1	126.92 (16)	O3—C11—C10	116.7 (2)
C7—N2—Cu1	114.34 (15)	N2—C12—C10	123.4 (2)
C13—N3—Cu1	108.16 (16)	N2—C12—H12	118.3
C13—N3—H3A	108 (2)	C10—C12—H12	118.3
Cu1—N3—H3A	114 (2)	N3—C13—C14	107.8 (2)
C13—N3—H3B	110 (2)	N3—C13—H13A	110.1
Cu1—N3—H3B	108 (2)	C14—C13—H13A	110.1
H3A—N3—H3B	109 (3)	N3—C13—H13B	110.1
C14—N4—Cu1	107.68 (16)	C14—C13—H13B	110.1
C14—N4—H4B	106 (2)	H13A—C13—H13B	108.5
Cu1—N4—H4B	114 (2)	N4—C14—C13	107.7 (2)
C14—N4—H4C	108 (2)	N4—C14—H14A	110.2
Cu1—N4—H4C	110 (2)	C13—C14—H14A	110.2
H4B—N4—H4C	110 (3)	N4—C14—H14B	110.2
N1—C1—C2	123.1 (2)	C13—C14—H14B	110.2
N1—C1—H1	118.5	H14A—C14—H14B	108.5
C2—C1—H1	118.5		
			
C1—N1—Cu1—N3	2.7 (2)	N2—C7—C8—C9	−0.3 (4)
C6—N1—Cu1—N3	−175.05 (17)	C6—C7—C8—C9	179.0 (2)
C1—N1—Cu1—N2	−178.1 (2)	C7—C8—C9—C10	−0.2 (4)
C6—N1—Cu1—N2	4.10 (17)	C8—C9—C10—C12	−0.3 (4)
C12—N2—Cu1—N4	−6.3 (2)	C8—C9—C10—C11	178.8 (2)
C7—N2—Cu1—N4	171.90 (17)	C9—C10—C11—O4	162.4 (2)
C12—N2—Cu1—N1	177.6 (2)	C12—C10—C11—O4	−18.6 (3)
C7—N2—Cu1—N1	−4.19 (16)	C9—C10—C11—O3	−18.6 (4)
C13—N3—Cu1—N4	13.46 (17)	C12—C10—C11—O3	160.4 (2)
C13—N3—Cu1—N1	−170.47 (17)	C9—C10—C12—N2	1.5 (4)
C14—N4—Cu1—N3	15.41 (18)	C11—C10—C12—N2	−177.6 (2)
C14—N4—Cu1—N2	−163.62 (17)	N3—C13—C14—N4	53.5 (3)
N1—C1—C2—C4	−1.4 (4)	C2—C1—N1—C6	1.8 (4)
N1—C1—C2—C3	−178.9 (3)	C2—C1—N1—Cu1	−175.9 (2)
C4—C2—C3—O1	−167.1 (3)	C5—C6—N1—C1	−0.5 (4)
C1—C2—C3—O1	10.4 (4)	C7—C6—N1—C1	178.7 (2)
C4—C2—C3—O2	11.9 (4)	C5—C6—N1—Cu1	177.44 (18)
C1—C2—C3—O2	−170.6 (3)	C7—C6—N1—Cu1	−3.3 (3)
C1—C2—C4—C5	−0.3 (4)	C10—C12—N2—C7	−2.0 (4)
C3—C2—C4—C5	177.2 (2)	C10—C12—N2—Cu1	176.16 (18)
C2—C4—C5—C6	1.4 (4)	C8—C7—N2—C12	1.4 (3)
C4—C5—C6—N1	−1.1 (4)	C6—C7—N2—C12	−178.0 (2)
C4—C5—C6—C7	179.8 (2)	C8—C7—N2—Cu1	−177.01 (18)
N1—C6—C7—N2	−0.2 (3)	C6—C7—N2—Cu1	3.6 (2)
C5—C6—C7—N2	179.0 (2)	C14—C13—N3—Cu1	−39.3 (2)
N1—C6—C7—C8	−179.6 (2)	C13—C14—N4—Cu1	−40.8 (3)
C5—C6—C7—C8	−0.4 (4)		

### Hydrogen-bond geometry (Å, °)

**Table:**

*D*—H···*A*	*D*—H	H···*A*	*D*···*A*	*D*—H···*A*
N3—H3A···O2^i^	0.84 (3)	2.11 (3)	2.881 (3)	153 (4)
N3—H3B···O3^ii^	0.86 (4)	2.21 (4)	3.031 (3)	159 (4)
N4—H4B···O8	0.87 (3)	2.20 (3)	3.054 (3)	171 (4)
N4—H4C···O9^iii^	0.89 (4)	2.23 (4)	3.069 (4)	158 (4)
O5—H5B···O7^iv^	0.86 (6)	1.97 (6)	2.807 (4)	164 (5)
O5—H5C···O9^ii^	0.72 (7)	2.08 (6)	2.779 (5)	165 (5)
O6—H6A···O1^i^	0.74 (5)	1.99 (5)	2.726 (4)	171 (4)
O6—H6B···O3^iii^	0.95 (7)	2.51 (7)	3.267 (4)	136 (5)
O7—H7A···O2	0.82 (6)	2.00 (6)	2.776 (5)	158 (5)
O7—H7B···O4^v^	0.78 (7)	2.11 (7)	2.827 (4)	153 (7)
O8—H8B···O7^iv^	0.76 (6)	2.22 (6)	2.969 (5)	178 (8)
O9—H9B···O4^vi^	0.86 (6)	1.96 (5)	2.744 (4)	152 (5)
O9—H9C···O3	0.97 (6)	1.74 (6)	2.706 (3)	169 (4)

Symmetry codes: (i) −*x*+1/2, *y*−1/2, −*z*+3/2; (ii) *x*, −*y*+2, *z*+1/2; (iii) *x*, −*y*+1, *z*+1/2; (iv) −*x*+1/2, −*y*+5/2, −*z*+1; (v) −*x*+1/2, −*y*+3/2, −*z*+1; (vi) −*x*, −*y*+1, −*z*.
